# Dust formation in French fries

**DOI:** 10.1016/j.crfs.2023.100466

**Published:** 2023-02-25

**Authors:** R.G.M. van der Sman, Bjorn van den Oudenhoven

**Affiliations:** a Wageningen Food & Biobased Research, the Netherlands; b Lamb-Weston Meijer, Kruiningen, the Netherlands

**Keywords:** Freezing, Frying, Stress, Fracturing

## Abstract

In this study we report on the analysis of dust formation, a quality problem arising in the industrial processing of par-fried, frozen french fries. This dust constitutes fractured pieces broken off the crust during finish frying. We claim that this dust problem has many similarities with flaking arising during the final-baking of par-baked french baguettes, i.e. the two problems are governed by the same physical principles. Inspired by the hypotheses behind flaking, we have made an experimental design, where we have perturbed the operating conditions of an industrial processing line of french fries. The measured dust during finish frying is correlated with the physical properties of the crust, measured in the different unit operations of the industrial processing line, and the operating conditions. We have shown that dust is non-linearly correlated to 1) the moisture content of the crust as influenced by drying and par-frying, and 2) the freezing rate in the industrial tunnel freezer. Remarkably, the amount of dust decrease with the increase of frozen storage time, which we have explained via viscoelastic relaxation of locked-in stress - mediated by moisture migrating from core to crust. This decay is shown to be independent of pretreatments, which only determines its starting value. With the given relations industry can in principle control the dust problem, but these measures have to be weighed against their effects on other objectives of the industry.

## Highlights

• Crust fracturing during finish frying (dust) is due to interaction between various unit operations.
• Dust is non-linearly related to moisture removal and freezing rate
• Crust remains largely unfrozen during freezing operations, subjecting it to stress build up.
• There is much similarity between dust and flaking of par-baked prefrozen french baguette.

## 1 Introduction

During the finish-frying of french fries, which are priorly par-fried and frozen, dust formation can occur. This so-called dust is composed of small fragments from the crust of the french fry, that detach via fracturing from the crust during the finish-frying. The dust floats on top of the frying oil, and it has to be skimmed off to prevent the fouling of the frying oil. The dust formation is viewed as a major quality problem by manufacturers of par-fried fries, but the reasons for this dust formation are still elusive. The industry is seeking a resolution to this quality problem via adjustments of processing parameters of the treatments on fries in their factory, which largely comprise slicing, blanching, drying, par-frying, cooling, and freezing.

A research program has been formulated to get an understanding of the dust formation problem. Experiments have been designed on hypotheses formulated for the so-called flake formation during the manufacturing of par-baked french baguettes (Le Bail et al., 2005), which bears large similarities with the dust problem of french fries. Indeed, the manufacturing of the par-baked baguettes has much in common with french fries manufacturing, involving par-baking, cooling, and freezing. Likewise dust, the flake formation becomes apparent only at the end of the production chain, at the final baking in the supermarket, or with the consumer. The problem of flake formation has been studied, and reported in the scientific literature in several papers (Lucas et al., 2005; Le Bail et al., 2005; Ribotta and Le Bail, 2007). For flaking, hypotheses have been formulated, which we assume also to hold for dust formation. Although flaking is apparent only in the final step of the production chain, its cause has been linked to prior unit operations in the production chain. Furthermore, it is thought that the interactions between conditions in various unit operations are affecting flake formation. It is reasonable to assume that it also holds for dust formation.

In our research program, we investigated dust formation via perturbations of the standard processing of french fries in the factory. In the first phase of the project, we investigated the perturbation of single unit operations only. In the second phase, we studied the interaction between unit operations, via simultaneous perturbation of two unit operations. Next, to the analysis of the resulting dust formation, we measured also several physical properties of samples taken from various steps in the production chain. These extra measurements helped us in validating and refining our hypotheses. After presenting the results, we discuss them in light of the first formulated hypotheses, which will be updated if required.

## 2 Hypotheses

### 2.1 Flake formation in par-baked bread

Crust flaking in par-baked french bread has been investigated intensively by LeBail and coworkers (Lucas et al., 2005; Le Bail et al., 2005; Ribotta and Le Bail, 2007). We view that the flaking bears large similarities with dust formation in french fries, and we think that similar hypotheses would explain both phenomena. Here, we review the hypotheses behind flaking, as formulated by LeBail and coworkers.

Crust flaking is a quality problem of par-baked french bread that occurs during the final baking. Due to damage to the crust already inflicted during the preprocessing, the thermal shock during final baking induces fracturing of the crust, resulting in the detachment of flakes from the bread. The main cause of the damage to the crust is the freezing step. If the freezing step is absent, flaking is not observed. The par-baked bread dough received by the freezing step has two regions with quite different physical properties. The core of the par-baked bread is wet and quite porous, while the crust region is relatively dry and dense. This difference in material properties makes the two regions respond differently to freezing. The wet core region will expand due to its significant ice formation during freezing. The dry crust region will hardly see ice formation, and even it has been observed to contract instead to expanding. These different deformations lead to the build-up of strong tensile stresses, which can not relax way in the dry/frozen crust. It is assumed that the dry crust is in the glassy state, due to the moisture removal either during the parbaking or during freezing via the ice formation. Glassy materials are characterized by long viscoelastic relaxation times, and thus the stresses can not relax away under practical time scales.

This build-up of tensile stresses can result in microcracks, which will grow further during the thermal shock of the final baking. This damage is even more aggravated by the buildup of ice just under the crust. The extra ice is formed due to the water transport from the hot wet core to the cool, dry crust. This water transport is also dependent on the temperature gradient between crust and core, indicating the importance of reducing the core temperature during cooling before freezing. The freezing of this ice on the interface of crust and core creates highly localized tensile stresses and microcracks, making it possible that the crust can detach from the core during the final baking.

The humidity level of the crust region is determined by the preprocessing before the freezing step, namely the proofing, par-baking, and cooling step. During the frozen storage, the moisture gradient between the crust and core will be retained. The amount of flaking can be controlled via an increase in the crust humidity, but that might impair other quality traits of the par-baked bread. Hence, a compromise must be found.

Similar fracturing problems are known to happen for other bakery products. During normal baking of bread, fractures can form during the cooling phase after baking (Hirte et al., 2013). Furthermore, in biscuits cracks are formed in the cooling phase after baking (Bernussi et al., 1998; Ahmad et al., 2001). All these scientific studies indicate that the fracturing is due to thermal stresses occurring during fast temperature changes. For biscuits it is found that the amount of cracks is linear with the moisture gradients between crust and crumb (Bernussi et al., 1998).

### 2.2 Hypothesis for dust formation

**Fig. 1:**
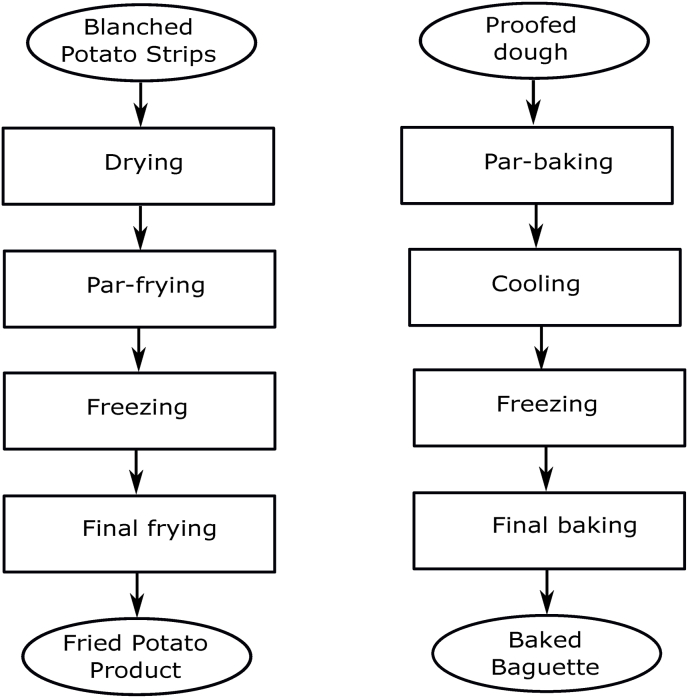
Production chain for french fries, in somewhat condensed form. We are concerned with drying, par-frying, and freezing. The incoming material is blanched sliced potato strips. The product chain of french fries is compared to that of par-baked baguettes, as shown on the right.

Inspired by the above hypotheses formulated for explaining flaking in par-baked bread, we formulate initial hypotheses concerning dust formation in par-fried french fries. We assume similar phenomena occur during similar steps in the production chain. In Fig. 1 we compare both production chains. Both par-baked bread, as well as par-fried fries, receive a final preparation via intensive heating (final baking or finish frying). Before this final step, the products have been actively frozen (with a belt freezer), and subsequently stored in the frozen state - which can take several weeks. To shorten the final preparation time during final baking/finish frying moisture is removed via par-baking or par-frying before the freezing step. To prepare the products for par-baking or par-frying they undergo proofing or blanching (for bread and fries respectively).

Likewise flaking, dust is formed during the final step of the production chain: the finish frying. Similar to final baking, we assume that the finish frying imposes a thermal shock to the crust, which causes the crust to fracture. Similar to par-baked bread, the crust has already been damaged in the preceding freezing step. Similar to par-baked bread, there is a significant moisture gradient between crust and core, leading to significant expansion of ice in the core region only. The difference in expansion leads to tensile stresses in the dry crust. Due to the reduced moisture content of the crust of the fry, we also think it will enter the glassy state during the freezing, and the developed tensile stresses can not relax away. Recently, long viscoelastic relaxation times have been established for starch (Van der Sman et al., 2022), the main constituent for both fries and bread. Due to the high elasticity of the glassy crust (Van der Sman et al., 2022), the frozen-in tensile stresses can only be dissipated via fracturing (van Vliet, 2002; Koç et al., 2013). Note that this dissipation of stress is rate dependent (i.e. the speed of how fast the external stress is applied, and the thus freezing rate). In a recent study, it is observed that mechanical damage indeed occurs during the processing of par-fried French fries, i.e. the frying and freezing (Vauvre et al., 2014). It is thought that ice formation makes cavities (pores) in the first two layers of cells (the crust). These cavities are often just below the surface. Connectivity of the pores to the surface is thought to be important for texture development and oil uptake during finishing frying. Hence, optimizing dust formation could imply compromising other quality traits of french fries.

In contrast to par-baked bread, we do not expect any extra buildup of ice at the crust/core interface, as the core of the french fry has very low porosity, prohibiting fast transport of moisture. In french fries, the ice formation happens both in the freezing step and the subsequent frozen storage. The freezing step of french fries is designed such that the core temperature will not exceed −12^*o*^C, and hence the ice crystal formation will be completed in the frozen storage at −18^*o*^C.

During the storage of the par-fried product in the frozen state, moisture can migrate from the core to the crust. This can induce a slight extra growth of ice crystals, promoting some extra crack formation in the crust. Initiators for these cracks might also be produced during the par-frying. It is assumed that the oil absorbed during the par-frying can act as a moisture barrier, hindering in some way the moisture migration from the core to the crust (Patent US8435583B2).

## 3 Materials and methods

Dust formation is investigated by perturbing the standard processing of french fries in the factory. The manufacturing of french fries is described in detail in (Agblor and Scanlon, 2000). The unit operations we will perturb are 1) drying, 2) par-frying, and 3) freezing. After freezing in the factory, the fries are stored, where they are frozen further down to −18^*o*^*C*. Before drying the fries (can) receive several pre-treatments like cutting, blanching, and soaking with SAPP (sodium acid pyrophosphate), salt and glucose.

**Fig. 2:**
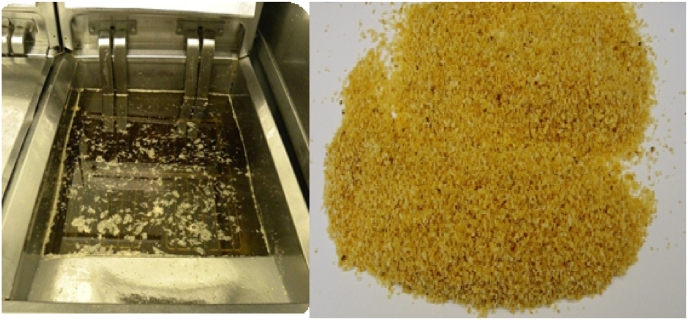
Example of dust generated in the fryer during finish frying. The floating dust is collected via skimming (right pane) and its weight has been measured.

Dust formation is measured after a certain period of frozen storage via final frying in a retail fryer using a standardized industrial protocol, resulting in the number of grams of dust per weight of the fried samples. The dust, being the solid particles floating in the frying oil, is collected by skimming the oil of the fryer with a fine sieve. Photos of (collected) dust in the finish fryer is shown in Fig. 2. Before, new measurements we ensure that frying oil is free of any dust, preventing build up of fines. For every new test, as described in Table 1, Table 2, new fresh oil is used.

**Table 1:** Single perturbations of the production process. * indicates samples used for experiments examining dust as a function of frozen storage time. ^†^ indicates standard treatments.

Dryer test			
Code	*T*_*dry*_ (^*o*^*C*)	*τ*_*dry*_ (*min*)	R.H. (%)
D1*	70	12	35
D2	65	11	38
D3	60	11	42
D4*^,†^	55	10	45
D5	50	9	48
D6*	40	8	55
Fryer test			
Code	Toil(Co)	*τ*_*fry*_ (*s*)	
Y1*	190	60	
Y2	187	57	
Y3	183	55	
Y4*^,†^	180	50	
Y5	177	47	
Y6	173	43	
Y7*	170	40	
Freezer test			
Code	Tfrz(Co)	Fan Speed (%)	
F1*	−14	100	
F2	−14	75	
F3	−11	100	
F4*^,†^	−11	75	
F5	−11	50	
F6	−8	75	
F7*	−8	50

**Table 2:** Multiple combined perturbations of the production process.

Dryer-Fryer test					
Code	Tdryer(Co)	*τ*_*dry*_ (*min*)	R.H. (%)	Toil(Co)	*τ*_*fry*_ (s)
DY1	70	12	35	190	60
DY2	65	11	38	185	55
DY3	65	10	42	176	45
DY4	45	9	52	180	51
DY5	57	8	48	173	43
DY6	40	8	55	170	40
Fryer-Freezer test					
Code	Toil(Co)	*τ*_*fry*_ (*s*)	Tfrz(Co)	Fan Speed (%)	
YF1	167	45	−20	50	
YF2	187	64	−20	100	
YF3	193	70	−12	100	
YF4	169	46	−12	50	
YF5	182	54	−14.5	100	
YF6	172	50	−14.5	50	
YF7	177	53	−17	50	
YF8	187	59	−17	100	

We note, our relative simple method of measuring dust might be prone due some systematic deviation from the true value of dust, as these particle can continue to loose moisture, or take up waxes or oxidized oil. As we keep frying time, temperature and mass of fries constant during the finish frying, we think the kinetics of these mass changes are similar, and only lead to systematic deviations. Consequently, we can still perform relative comparisons between different treatments.

To be able to link the dust formation to physical changes happening during a particular processing step, we have measured also temperature and moisture content at various points in the production chain. To this end, we have split the fries into a crust region and a core region via a custom-made slicing apparatus. The crust region has a width of 1 mm and is taken from two sides of the fry. For both crust and core, the temperature and moisture content is measured. Moisture content (dry matter) is determined via a standard drying oven test. However, after frying the crust also contains fat, and hence both fat content and (hydrophilic) dry matter are distinguished. Furthermore, to be able to determine the freezing behaviour of the crust, its salt and sugar content has been measured.

The various perturbations applied to the standard process are indicated in Table 1, Table 2 In the dryer test, we have varied the drying temperature, *T*_*dry*_, the dryer residence time, *τ*_*dry*_, and the relative humidity of the drying air *R*.*H*. In the fryer test, we have varied the frying oil temperature *T*_*oil*_, and the fryer residence time *τ*_*fry*_. In the freezer test, we have varied the freezer temperature *T*_*frz*_ and fan speed, via which we have varied the freezing rate. At the beginning of the project, we perturbed single processing, and later we investigated also interactions between several processing steps, as indicated in Table 2. The other unperturbed processes are kept at standard operating conditions, which are indicated in Table 1 with ^†^. Two combined tests were performed, varying process parameters of a) both dryer and fryer and b) both fryer and freezer. Note, that during all tests the blanching conditions are kept the same as during industrial processing. The perturbations are designed such that we obtain maximal differences between treatments, while still remaining within bounds set by the manufacturer. Consequently, we hope to get significant differences between treatments, enabling us to perform correlative analysis.

After the processing, the fries are put in frozen storage, conditioned at −18^*o*^C. Samples are taken multiple times from the frozen storage, and the amount of dust is determined via our standard method using finish-frying. Five independent samples are taken for dust determination per treatment. Using the values of all samples we computed the average value and the standard error, using a 95% confidence interval.

## 4 Results

**Fig. 3:**
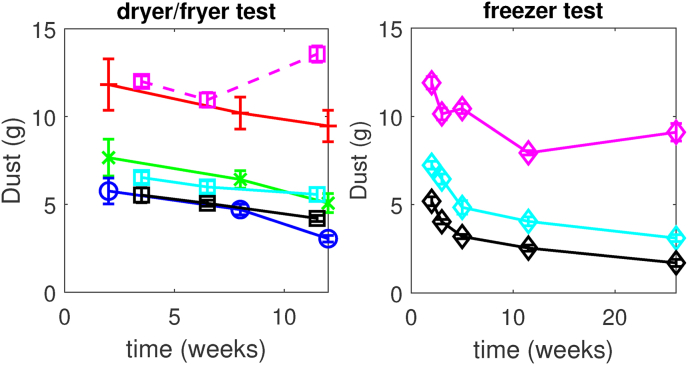
Amount of dust against frozen storage time for different experiments in a) dryer and fryer tests (’r+’ = D1, ’gx’ = D4, ’bo’ = D6, ’ms' = Y1, ’cs' = Y4, ’ks' = Y7), and b) freezer test (’md’ = F1, ’cd’ = F4, ’kd’ = F7). Symbols within quotes use Matlab plotting codes, and the other codes refer to treatments as defined in Table 1. The solid lines are intended to guide the eye. Error bars indicate standard errors.

For some of the dryer, fryer, and freezer tests the dust formation is followed as a function of (frozen) storage time (these samples are indicated with an asterisk in Table 1). These results are presented in Fig. 3. In the left pane, we observe that for most tests the amount of dust decreases with storage time at a comparable, and constant rate. There is one outlier for dust after 12 weeks of storage after the fryer test Y1. The right pane shows that the decrease of dust with storage time for the freezer experiment appears to decay at a comparable rate as for the other treatments, as shown in Fig. 3. However, in the first 2 weeks after the freezer test, the dust formation shows a faster decrease, more consistent with exponential decay.

This observation of a decrease of dust with storage time invalidates the hypothesis that during frozen storage there is moisture migration from core to crust, leading to an increase of ice growth and dust. We think that this decrease in dust with storage time is the result of the viscoelastic relaxation of the frozen-in stresses. At the time scale of 12 weeks of frozen storage, the viscoelastic relaxation in the crust is sufficiently fast for a significant reduction of dust. The exponential decay of dust observed after the freezer test is consistent with viscoelastic relaxation, as follows from the commonly used Maxwell model.

Here, we pose that at constant frozen storage conditions, the evolution of dust with storage time follows a similar exponential decay and that only the initial value is determined by the pretreatments before storage. Hence, to compare the effects of different pretreatments on dust, we can compare their dust values at any given storage time. Below, we continue our analysis where we investigate correlations of dust with physical parameters measured on the fries during/after pretreatments.

**Fig. 4:**
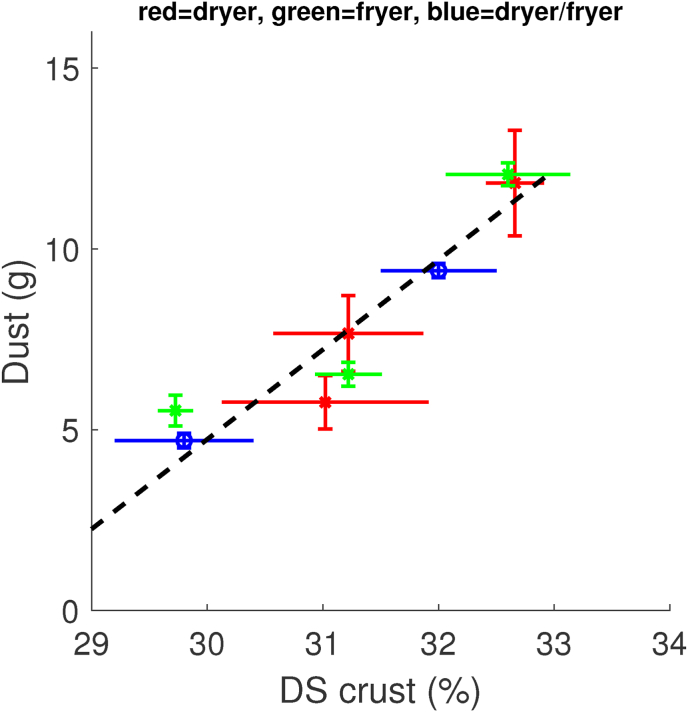
Amount of dust against the relative amount of dry solids (DS) in the crust region after par-frying. Data is taken from the dryer test (red), fryer test (green), and combined dryer-fryer test (blue). The dashed line indicates the result of linear regression, showing *r*^2^ = 0.75. (For interpretation of the references to colour in this figure legend, the reader is referred to the Web version of this article.)

The objective of the drying and frying processing steps is to remove moisture from the crust region. From our measurements, we have determined the dry matter content of the crust (denoted as *DS*) of samples taken directly after the dryer or fryer treatments. We note, that *DS* is excluding the fat, which has been determined separately. Consequently, 1 − *DS* can be viewed as a measure for the amount of moisture removed from the crust. These *DS* values are compared to the amount of Dust determined from samples taken from frozen storage after 3 weeks, with results shown in Fig. 4. Note, that we have also included data from the combined dryer/fryer test. The level of DS in the crust ranges from 29 to 34%, which was significantly higher than moisture content of the core, which largely remained constant over processing up to par-frying. Measurements show that the value for the core was about *DS* = 26.2 ± 0.4%. Hence, par-frying creates a moisture gradient over the crust. We have also performed linear regression to determine the best-fitting line between the data points. This indicates a good correlation between DS of the crust and Dust.

**Fig. 5:**
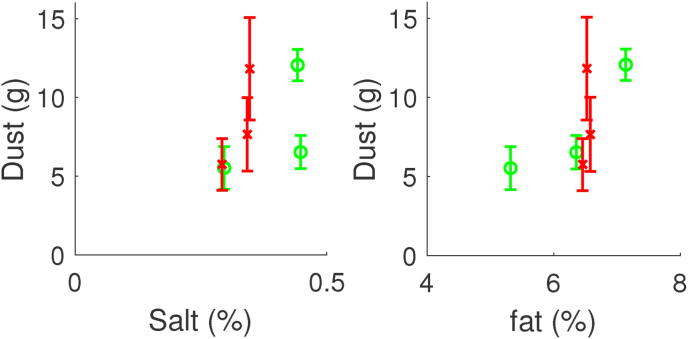
Amount of dust against the relative amount of salt and fat in the crust region after par-frying. Data is taken from the dryer test (red symbols), and fryer test (green symbols). (For interpretation of the references to colour in this figure legend, the reader is referred to the Web version of this article.)

For the dryer and fryer tests, we have also investigated correlations of Dust with other physical parameters, such as the salt content and the fat content of the crust. These correlations are shown in Fig. 5. As one can observe, there is no clear correlation between Dust with either fat or salt content.

**Fig. 6:**
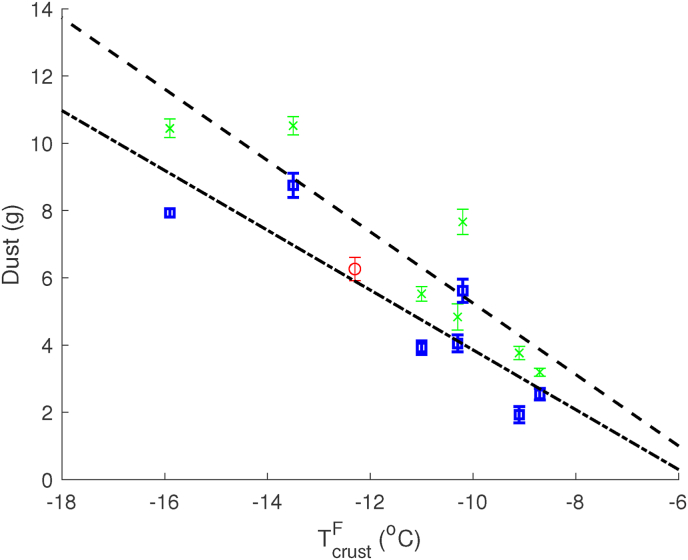
Effect of freezing rate, as reflected in crust temperature after freezing, on dust formation. Via regression, a linear trend line can be drawn through the experimental data, for 5 weeks (green) and 12 weeks (blue) respectively. The red symbol is the result of the dryer/fryer test. Standard errors on crust temperature are small Δ*T*_*crust*_ ≤ 0.4^*o*^*C*, and therefore they are not drawn in the graph. Regression analysis renders a *r*^2^ = 0.66 and 0.59 for data of 5 weeks and 12 weeks of storage. (For interpretation of the references to colour in this figure legend, the reader is referred to the Web version of this article.)

During the freezer test, we varied the freezing rate and analysed its effect on Dust. During the freezer test, we measured the crust temperature of fries leaving the freezing tunnel with handheld infrared sensors. Because the residence time was the same for all treatments in the freezer test, the crust temperature, $T_{\mathrm{crust}}^{F}$, can be taken as an indicator of the freezing rate. In Fig. 6 we have plotted Dust against $T_{\mathrm{crust}}^{F}$, with Dust measured after 5 and 12 weeks after the freezer test. In the figure, we observe a linear correlation between crust temperature and dust formation. In Fig. 6 we have also added an extra data point from the fryer test having the same pretreatment as the fries in the freezer experiment. This data point is indicated in red in Fig. 6 and shows to be consistent with the freezer experiment. Comparing Dust after 5 and 12 weeks indicates again a decrease over time. The similar slopes of the regression lines indicate again that this decrease is largely independent of pretreatments.

From the results presented in the above figures, we can conclude that the amount of dust depends on three physical factors: a) the amount of dry solids (DS) in the crust, b) the crust temperature after freezing $T_{\mathrm{crust}}^{F}$ (i.e. freezing rate), and c) the storage time. For all experiments, we have collected these data. Via multiple linear regression, we investigate how far these factors explain the observed variation of dust. Preliminary analysis using a simple linear regression model with the above three parameters indicates that dust is not well explained by a simple linear model. Regression analysis shows that only a *r*^2^ = 0.42 is achieved. The exponential decay of dust during frozen storage is another indication that a linear model will not give a good description.

**Fig. 7:**
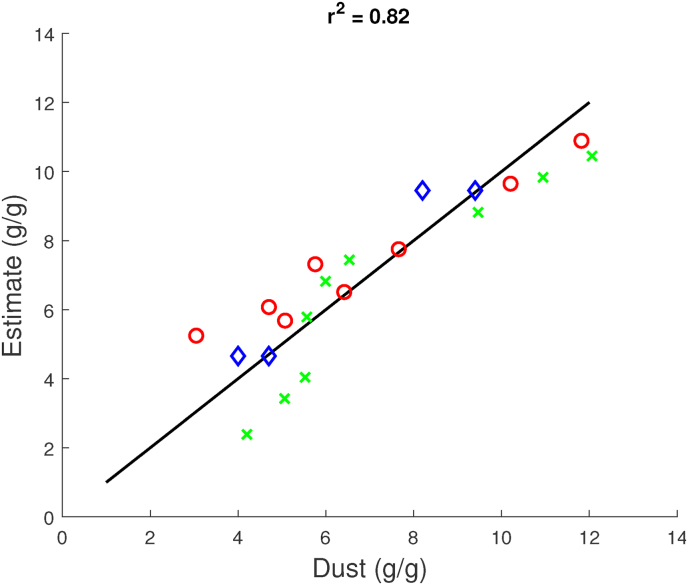
Observed Dust values versus predicted dust values, according to the multi-linear regression model, using only data from the dryer (red), fryer (green), and combined dryer/fryer (blue) tests. The linear regression model, involving *DS* and storage time only, indicates a *r*^2^ = 0.82. (For interpretation of the references to colour in this figure legend, the reader is referred to the Web version of this article.)

**Fig. 8:**
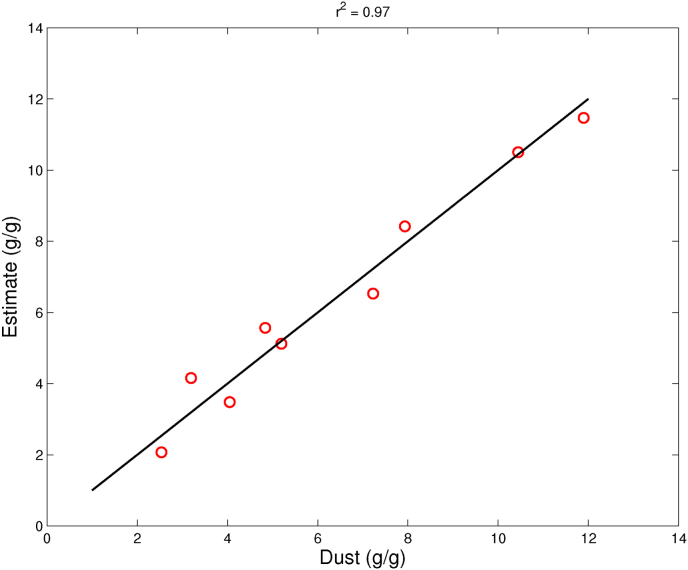
Observed Dust values versus predicted dust values, according to the multi-linear regression model, using only data from the freezer test. The linear regression model, involving *T*_*crust*_ and storage time only, indicates a *r*^2^ = 0.97.

The correlations improve if 1) we include only data on the fryer and dryer test, where *DS* and storage time are the two explaining physical factors, or 2) we include only data on the freezer test, where $T_{\mathrm{crust}}^{F}$ and storage time are the two explaining physical factors. The results are shown in Fig. 7, Fig. 8. Now, the regression coefficients are *r*^2^ = 0.75 and *r*^2^ = 0.97. Hence, the poor correlation of the preliminary regression analysis can be explained by the probable cause that *DS* and $T_{\mathrm{crust}}^{F}$ are **not** independent variables.

**Fig. 9:**
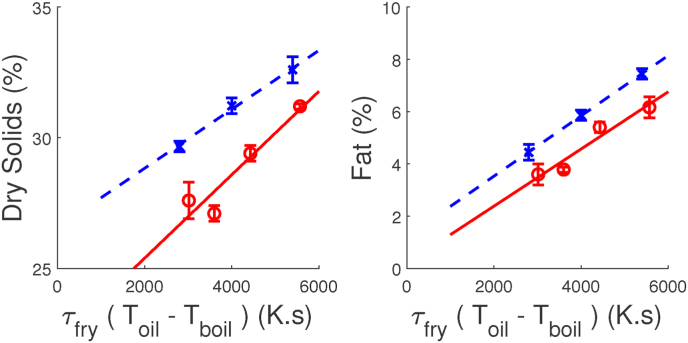
Energy input during frying, *τ*_*fry*_(*T*_*oil*_ − *T*_*boil*_) can be related to a) DS of the crust after frying (left pane) and b) fat content of the crust after frying (right pane). Data are used from the fryer test (blue symbols) and the combined fryer/freezer (red symbols). The lines are the results of linear regression, with *r*^2^ > 0.8. (For interpretation of the references to colour in this figure legend, the reader is referred to the Web version of this article.)

**Fig. 10:**
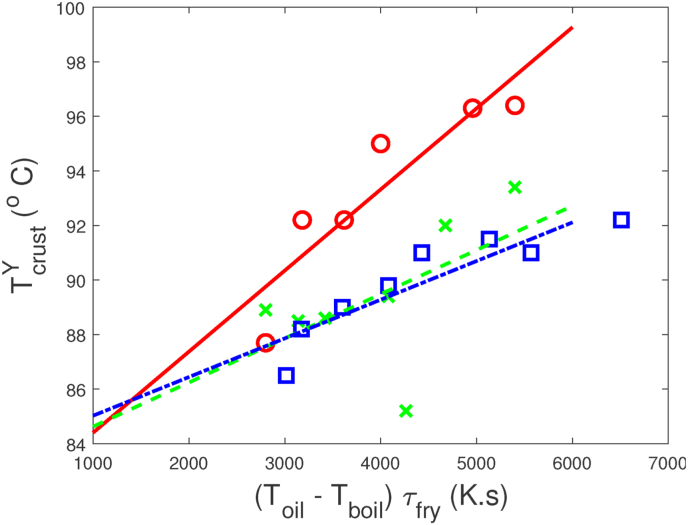
Crust temperature after frying as function of energy input during frying, as represented by (*T*_*oil*_ − *T*_*boil*_)*τ*_*fry*_. Data are taken from the fryer test (red), combined dryer/fryer (green), and combined fryer/freezer (blue). The lines are the results of linear regression, with *r*^2^ ≥ 0.72. (For interpretation of the references to colour in this figure legend, the reader is referred to the Web version of this article.)

After establishing correlations between dust and the physical properties of the crust, we need to link them to processing conditions. We investigate how fryer processing conditions can be linked to DS of the crust. DS is determined by the amount of moisture removed during frying, which is related to the amount of heat supplied by the frying oil to remove this moisture. A simplified energy balance indicates that this energy should be linear with *E* = *τ*_*fry*_(*T*_*oil*_ − *T*_*boil*_), with *τ*_*fry*_ the residence time of fries in the fryer, *T*_*oil*_ the oil temperature, and *T*_*boil*_ is the boiling point of water. In Fig. 9 we show the correlation between *DS* and *E*, with error bars based on the standard error. Additionally, we have correlated fat content with frying energy *E*, as shown in the right pane of Fig. 9, confirming the hypothesis from the literature that the porous space in the crust created by the removal of moisture during frying, will be (partially) filled by fat upon removal of the fries from the (par-frying) oven (Vauvre et al., 2014). Furthermore, the energy input *E* we have correlated with the crust temperature after frying, $T_{\mathrm{crust}}^{Y}$, with results shown in Fig. 10. We observe indeed a linear correlation between *E* and $T_{\mathrm{crust}}^{Y}$, but the gradient of the line is different for the fryer test (in red). The crust temperature is taken out of fryer for the measurement using an infrared thermometer. The fryer test was performed on another day than the combined trials. Hence, we attribute the difference in gradient due to differences in environmental conditions, leading to slower cooling during the fryer test. In our further analysis we use *E* as an explaining parameter, which is independent of environmental conditions.

**Fig. 11:**
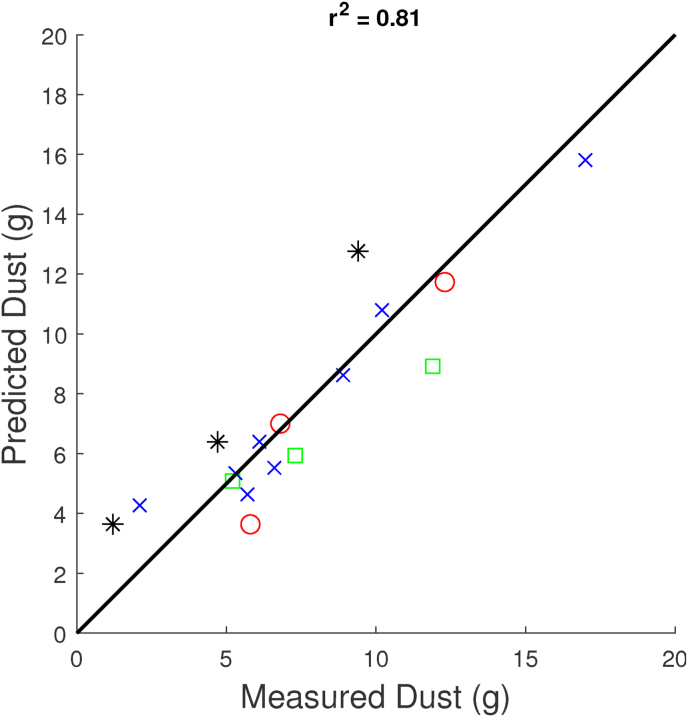
Observed Dust values versus predicted dust values, according to the non-linear regression model with FryingEnergy and Frozen crust temperature as explaining variables. Experimental data from various tests. The non-linear model Eq. (1) renders *r*^2^ = 0.81. Red symbols relate to results from the fryer test, green symbols relate to results from the freezer test, black symbols indicate results from the combined dryer/fryer test, and blue symbols indicate results from the combined fryer/freezer test. (For interpretation of the references to colour in this figure legend, the reader is referred to the Web version of this article.)

We analyse further the interaction between freezing and frying on dust formation, using fryer energy input *E* = (*T*_*oil*_ − *T*_*boil*_)*τ*_*fry*_ and $T_{\mathrm{crust}}^{F}$ (after freezing) as explaining variables, using Dust measured after 3 weeks. Results are shown in Fig. 11.

**Fig. 12:**
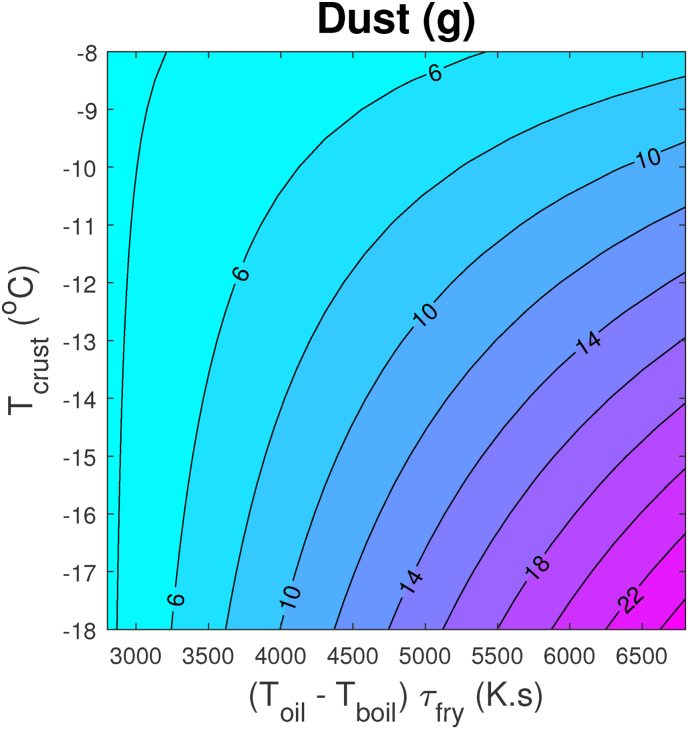
Counterplot for dust as function of energy input during frying (linear with *E* = *T*_*oil*_ − *T*_*boil*_)*τ*_*fry*_, and crust temperature during freezing $T_{\mathrm{crust}}^{F}$. The plot is obtained from the fitted non-linear model, Eq. (1).

Statistical analysis shows that the experimental data can be explained by the following non-linear regression model:

(1) $$Dust=a_{0}+a_{1}E+a_{2}\left(T_{\mathrm{crust}}^{F}-T_{0}\right)+a_{3}E\times T_{\mathrm{crust}}^{F}$$

with *T*_0_ the freezing point of pure water. The fitting shows that *a*_0_ = 11.0, *a*_1_ = −2.6 × 10^−3^ (g/K.s), *a*_2_ = 1.24 (g/K), and *a*_3_ = −0.4 × 10^−3^ (*g*/*K*^2^.*s*). The *r*^2^ value is *r*^2^ = 0.81. To indicate the non-linear relation, we show a contour plot of Dust versus *E* and $T_{\mathrm{crust}}^{F}$ in Fig. 12.

**Fig. 13:**
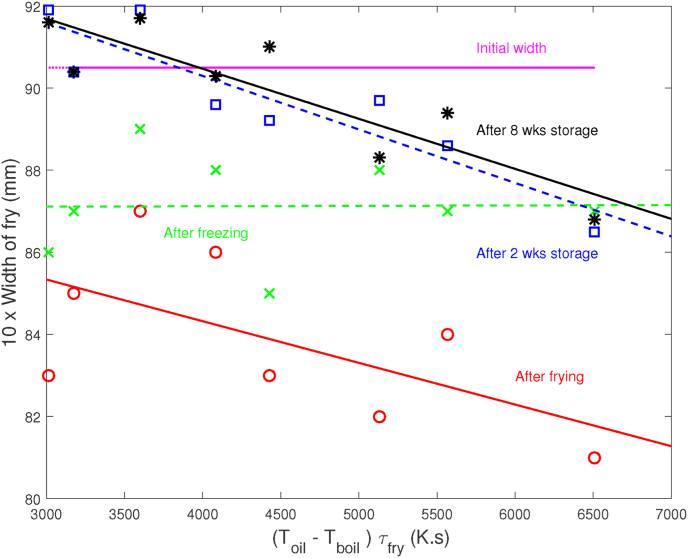
Width of the fry as function of energy input during frying (linear with (*T*_*oil*_ − *T*_*boil*_)*τ*_*fry*_, sampled at different moments during processing. Lines are obtained via linear regression of the experimental data.

During the combined fryer/freezer test, we also measured the width and mass of the fries, at different points in the production chain (Before and after drying, after frying, after freezing, and after 2 and 8 weeks of frozen storage) to examine possible locations where the crust can experience mechanical stresses. Results are shown in Fig. 13. After drying the width is about equal to the initial width of 90.5 mm. The thickness after frying is linear with the frying energy input, which complies with previous findings concerning dry solids (DS) and the fat content of the crust. The data after freezing has too much scatter for drawing any conclusions. The thickness after frozen storage is again linear with the frying energy input. However, after 2 weeks of frozen storage - the fry does not expand anymore. The expansion of the fry during frozen storage is due to the thermal expansion of ice being formed during the freezer step and frozen storage. The extra expansion during frozen storage appears to be independent of frying energy, as is evident by the nearly identical slopes of the regression lines in Fig. 13. The (constant) frozen storage conditions determine only this constant extra expansion.

## 5 Discussion

We have found that dust formation is dependent on three factors: a) the amount of moisture in the crust after frying, b) the freezing rate, and c) frozen storage time. For all experiments, it holds that dust decreases with the duration of the frozen storage, with a rate rather independent of the pre-treatments, which thus only determines the initial value at the start of the frozen storage. We discuss the effect of pretreatments only concerning this initial value of dust.

The effects of dryer and fryer treatments are very similar regarding dust, as they both lower the amount of moisture in the crust region. The amount of moisture removed during frying is well controlled via the amount of thermal energy *E* ∼ (*T*_*oil*_ − *T*_*boil*_)*τ*_*fry*_ transferred during frying. At similar dry matter content of the crust (*DS*), the freezing rate is well characterized by the crust temperature after freezing $T_{\mathrm{crust}}^{F}$. Changes to *DS* will change the initial freezing point of the crust, and thus the freezing rate (as described by the Planck equation (Pham, 1984; Van der Sman et al., 2013)), and also $T_{\mathrm{crust}}^{F}$ - even at the same freezing conditions. This interaction explains why a non-linear regression model must be used to explain dust in terms of *DS* and $T_{\mathrm{crust}}^{F}$. The dimensions of the fry shrink after frying, but they increase again during freezing and frozen storage due to ice formation. These deformations will impart stresses on the dry crust, which relaxes away only very slowly, due to the dryness of the crust and low temperature. In a previous publication, we have indeed shown that the logarithm of the viscoelastic relaxation time of starch (the main component in fries) is more or less linear with *T*_*g*_/*T*, with *T*_*g*_ the moisture-dependent glass transition temperature (in Kelvin) (Van der Sman et al., 2022).

**Fig. 14:**
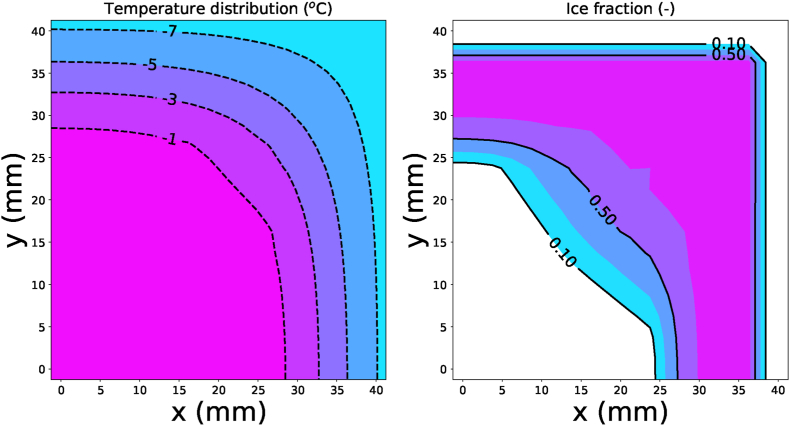
Contour plots of the temperature and ice fraction distribution in the cross-section of a par-fried french fry after freezing pretreatment. The origin is at the center of the cross-section.

From visual observations, we had the impression that the crust region of the fry remains unfrozen after the freezer treatment. To test this hypothesis, we have developed a simulation model for the freezing of french fries, taking into account the drying and par-frying pre-treatments (van der Sman, 2022). These pre-treatments resulted in lower moisture content of the crust region, compared to the core region - which we have taken as an initial condition for the freezer model. Furthermore, we have assumed that salt taken up from the brine pre-treatment is only taken up by the crust region. The uptake of solutes, combined with the moisture removal by drying and par-frying in the crust region has lowered its initial freezing point. The description of the numerical model will be given in a separate companying paper. Fig. 14 shows the computed temperature and ice distribution after 15 min of freezing, after drying and frying at standard conditions. We observe that the crust region and the center of the core are still unfrozen. The crust region is still free of ice due to its lower moisture content, and the solutes present. The crust's initial freezing point is shown to be around −10^*o*^C. Hence, only during frozen storage, some ice crystals will be formed in the crust region, but their amount will be significantly lower than the ice fraction in the core region.

Hence, during the freezing step ice expansion will occur only in the core region. This expansion will stretch the dry crust region, probably imposing some tensile stresses on the crust (Shi et al., 1998; Pham et al., 2005). The ice formation, expansion, and stretching of the crust will be continued in the first days of the frozen storage until the french fries are fully frozen at −18^*o*^C.

With our experimental results, we have re-evaluated our above hypotheses (from section 2.2) and the similarity of dust formation with crust flaking in par-baked baguettes. We have observed that the dust formation is indeed happening during finish frying due to the fracturing of the crust. Similar to parfrying, the crust will shrink during finish frying, imparting tensile stresses on the crust - leading to fracturing as a means to dissipate all the mechanical stresses accumulated in the crust during processing Luyten and Vliet (2006). This is due to a) crust shrinkage during par-frying, and b) core expansion and subsequent stretching of the unfrozen crust during the freezing step Le Bail et al. (2005); Lucas et al. (2005). These accumulated stresses will not have relaxed away during frozen storage. We assume that the fracturing during finish frying is initiated at microcracks, which have already formed during the par-frying or freezing step (Achir et al., 2010). The amount of accumulated stress depends on the magnitude of the elastic modulus, which increases with the lowering of moisture content - in a similar way as starch (Van der Sman et al., 2022).

The scientific literature on cryo-injury suggests this mechanism too (Steif et al., 2007). It is stated that during freezing thermal stresses only develop if the temperature drops down the ”set temperature”, corresponding to a viscosity of *η* = 10^7^ Pa s (Steif et al., 2007). Similar phenomena we have found earlier for the expansion of starchy snacks, where the matrix only yields if this critical viscosity is exceeded (van der Sman and Broeze, 2014). Our recent paper on the rheology of starch and maltodextrins suggests that long relaxation times are only developed at sufficiently large viscosities - which are shown to scale with *T*_*g*_/*T*, with *T*_*g*_ the moisture-dependent glass transition temperature (Van der Sman et al., 2022).

During frozen storage there is indeed no extra ice growth directly under the crust due to moisture migration - as evident by the constant size of the fry after two weeks of frozen storage. This aspect makes the dust formation different from the flaking problem, as is explained by the low porosity of the core of the french fry. The moisture migration during frozen storage probably does lead to viscoelastic relaxation of the locked-in stresses, and thus to a reduction of dust with storage time.

Within the framework of our current hypotheses, it is difficult to position the possible role of the type of fat used during the par-frying for dust formation. Previously, it is thought that fat crystals hinder moisture migration from core to crust, and it could reduce dust formation. However, in our current understanding reduction of moisture migration would even hinder the viscoelastic relaxation, and will keep dust formation at a high level. Perhaps, frying oil can have a similar role as shortening fat in bakery products, which makes the dough matrix softer, and thus less prone to fracturing. Yang et al. (2019) have investigated the possible role of different frying oils in different stages of industrial frying. They concluded that it is indeed difficult to pinpoint what determines the response of the different oil, but they also suggested it might be due to the differences in strength of the finish-fried crust.

This research will provide the industry with tools to reduce the dust problem: via a) an increase of moisture content of the crust via reduced par-frying, b) slower freezing, or c) longer frozen storage time. One must also be aware of the strong interaction between par-frying and freezing treatments. Dust is thus a prime example of a quality problem that needs consideration of the complete production chain (Yang et al., 2019), as we also discussed earlier in our review on the quality of frozen vegetables and fruits (Van der Sman, 2020). Other processing steps like blanching will also influence the mechanical strength of the cell wall material (due to possible action of PME or calcium, degradation of pectin, and gelatinization/retrogradation of starch).

However, these measures for reducing dust can compromise other objectives of the industry such as a short preparation time during finish frying, or the throughput of the fries in the factory. Furthermore, a wet crust can also promote clumping - which is another quality problem of frozen vegetables and fruits (Van der Sman, 2018). Hence, the industry needs to find a good balance concerning these different objectives.

## 6 Conclusions

In this paper, we have investigated the causes of dust formation during the finish-frying of frozen, par-fried french fries. Based on hypotheses formulated earlier for the problem of flaking in par-baked baguettes, we have constructed an experimental design, where we perturbed the regular production chain of french fries. It is shown that all steps in the production chain contribute to the amount of dust formed during finish frying. The most important physical factors contributing to dust are: a) the moisture content of the crust (as imparted by drying and par-frying), b) the freezing rate, and c) frozen storage time. However, we have noted a strong interaction between the first two physical factors, as lowering the moisture of the crust will determine its initial freezing point, and consequently the freezing rate. With the increase of frozen storage time, we have found a decrease in dust formation - which we have explained via viscoelastic relaxation, enabled by moisture migration from core to crust. The decay rate of dust during frozen storage is independent of the pretreatments, which thus only determines the start value of dust. For this starting value we have proposed a non-linear regression model, depending on moisture content (*DS*) and freezing rate (as indicated by $T_{\mathrm{crst}}^{F}$. Our results provide the industry with sufficient tools to reduce the dust problem. But, one should mind that such measures might impact other objectives of the industry.

## CRediT authorship contribution statement

**R.G.M. van der Sman:** Writing – original draft, wrote the manuscript, and designed the experimental plan. **Bjorn van den Oudenhoven:** coordinated the experiments at the processing plant of LambWeston in Kruiningen.

## Declaration of competing interest

The authors declare that they have no known competing financial interests or personal relationships that could have appeared to influence the work reported in this paper.

## Data Availability

Data will be made available on request.
